# Assessment of Health Information Technology–Related Outpatient Diagnostic Delays in the US Veterans Affairs Health Care System: A Qualitative Study of Aggregated Root Cause Analysis Data

**DOI:** 10.1001/jamanetworkopen.2020.6752

**Published:** 2020-06-25

**Authors:** Lauren Powell, Dean F. Sittig, Kristin Chrouser, Hardeep Singh

**Affiliations:** 1 Veterans Affairs (VA) National Center for Patient Safety, Ann Arbor, Michigan; 2 School of Biomedical Informatics, The University of Texas Health Science Center at Houston; 3 Department of Urology, University of Michigan, Ann Arbor; 4 Center for Innovations in Quality, Effectiveness, and Safety (IQuESt) at the Michael E. DeBakey VA Medical Center and Baylor College of Medicine, Houston, Texas

## Abstract

**Importance:**

Diagnostic delay in the outpatient setting is an emerging safety priority that health information technology (HIT) should help address. However, diagnostic delays have persisted, and new safety concerns associated with the use of HIT have emerged.

**Objective:**

To analyze HIT-related outpatient diagnostic delays within a large, integrated health care system.

**Design, Setting, and Participants:**

This cohort study involved qualitative content analysis of safety concerns identified in aggregated root cause analysis (RCA) data related to HIT and outpatient diagnostic delays. The setting was the US Department of Veterans Affairs using all RCAs submitted to the Veterans Affairs (VA) National Center for Patient Safety from January 1, 2013, to July 31, 2018.

**Main Outcomes and Measures:**

Common themes associated with the role of HIT-related safety concerns were identified and categorized according to the Health IT Safety framework for measuring, monitoring, and improving HIT safety. This framework includes 3 related domains (ie, safe HIT, safe use of HIT, and using HIT to improve safety) situated within an 8-dimensional sociotechnical model accounting for interacting technical and nontechnical variables associated with safety. Hence, themes identified enhanced understanding of the sociotechnical context and domain of HIT safety involved.

**Results:**

Of 214 RCAs categorized by the terms *delay* and *outpatient* submitted during the study period, 88 were identified as involving diagnostic delays and HIT, from which 172 unique HIT-related safety concerns were extracted (mean [SD], 1.97 [1.53] per RCA). Most safety concerns (82.6% [142 of 172]) involved problems with safe use of HIT, predominantly sociotechnical factors associated with people, workflow and communication, and a poorly designed human-computer interface. Fewer safety concerns involved problems with safe HIT (14.5% [25 of 172]) or using HIT to improve safety (0.3% [5 of 172]). The following 5 key high-risk areas for diagnostic delays emerged: managing electronic health record inbox notifications and communication, clinicians gathering key diagnostic information, technical problems, data entry problems, and failure of a system to track test results.

**Conclusions and Relevance:**

This qualitative study of a national RCA data set suggests that interventions to reduce outpatient diagnostic delays could aim to improve test result management, interoperability, data visualization, and order entry, as well as to decrease information overload.

## Key Points

**Question:**

What can be learned from analyzing health information technology–related outpatient diagnostic delays in a large, integrated health care system?

**Findings:**

In this cohort study of 214 root cause analyses, aggregated root cause analysis data involving health information technology and outpatient diagnostic delays in the Department of Veterans Affairs from 2013 to 2018 suggest that most safety concerns (83%) involved problems with safe use of technology, which were predominantly attributable to sociotechnical factors associated with people, workflow and communication, and a poorly designed human-computer interface.

**Meaning:**

This study suggests multiple interventions may be used to address outpatient diagnostic delays through improved design, configuration, and use of health information technology.

## Teaser

This qualitative study uses aggregated root cause analysis data to identify themes associated with health information technology–related outpatient diagnostic delays within a large, integrated health care system.

## Introduction

Diagnostic delays are a major threat to outpatient safety.^1^ Health information technology (HIT) can reduce diagnostic delays by reliably transmitting and tracking test results, supporting intelligent test selection, improving information access and display, and facilitating electronic communication.^2,3^ However, problems persist despite electronic health record (EHR) implementation, and new unintended safety concerns have emerged, spurring efforts to understand the consequences of HIT on diagnosis.^2,4,5,6,7,8,9,10,11,12,13^

For instance, inadequate test result follow-up is a substantial cause of diagnostic delays in EHR-enabled settings.^14^ Although electronic test result transmission is more reliable than one on paper, action on test results may be delayed.^15,16,17,18,19,20,21,22,23^ More than one-third of patients with lung cancer experience diagnostic delays, mostly from delayed follow-up of abnormal imaging findings.^24^ Similar follow-up failures can occur in bladder, gastrointestinal, and breast cancer diagnosis.^25,26,27,28^

The use of EHRs enables electronic notification of test results directly to clinicians’ inboxes. However, primary care practitioners (PCPs) receive excessive notifications that increase the risk of failing to see key information: about one-third of PCPs admit to missing abnormal test results.^29,30^ Many PCPs spend more than 1 hour daily on inbox management alone.^31,32,33^

Addressing diagnostic delays in the context of HIT requires improved understanding of complex systems that accounts for interactions between technology, its users, involved workflows, and organizational policies and procedures.^34^ In this study, we used the analytic lens of the Health IT Safety framework^6^ to generate a better understanding of diagnostic delays in the setting of HIT. The Health IT Safety framework provides a conceptual foundation for measuring, monitoring, and improving HIT safety and includes 3 related domains situated within an 8-dimensional sociotechnical model accounting for interacting technical and nontechnical variables associated with safety (Table 1).^7^ These domains include (1) safe HIT, (2) safe use of HIT, and (3) using HIT to improve safety (Table 2).^6^ We applied the Health IT Safety framework to a database of aggregated root cause analyses (RCAs) conducted within the Veterans Affairs (VA) health care system to characterize the role of HIT safety in outpatient diagnostic delays and to lay a foundation for potential solutions.

**Table 1.** Eight Sociotechnical Dimensions Guiding Data Analysis^a^

Sociotechnical dimension	Description
Hardware and software	Computing infrastructure used to support and operate clinical applications and devices
Clinical content	The text, numeric data, and images that constitute the language of clinical applications, including clinical decision support
Human-computer interface	All aspects of technology that users can see, touch, or hear as they interact with it
People	Everyone who is involved with patient care and/or interacts in some way with health care delivery (including technology). This would include patients, clinicians and other health care personnel, IT developers and other IT personnel, and informaticians
Workflow and communication	Processes to ensure that the information required for patient care is communicated effectively, efficiently, and safely
Internal organization features	Policies, procedures, the physical work environment, and the organizational culture that govern how the electronic health record system is configured, who uses it, and where and how it is used
External rules and regulations	Federal or state rules (eg, HIPAA and Meaningful Use) and billing requirements that facilitate or constrain the other dimensions
System measurement and monitoring	Evaluating both intended and unintended consequences through a variety of prospective, retrospective, quantitative, and qualitative measurements

Abbreviations: HIPAA, Health Insurance Portability and Accountability Act; IT, information technology.

^a^ Based on the 8-dimensional sociotechnical model by Sittig and Singh.^7^

**Table 2.** Health Information Technology (HIT) Safety Domains Guiding Data Analysis^a^

Domain	Principles
Safe HIT (address safety concerns unique to technology)	Data availability: HIT is accessible and usable on demand Data integrity: HIT data or information is accurate and has not been altered or destroyed in an unauthorized manner Data confidentiality: HIT data or information is only available or disclosed to authorized persons or processes
Safe use of HIT (optimize safe use of technology)	Complete/correct HIT use: HIT features and functionality are implemented and used as intended HIT system usability: HIT features and functionality are designed and implemented so that they can be used effectively, efficiently, and to the satisfaction of the intended users to minimize the potential for harm
Using HIT to improve safety (use technology to monitor and improve safety)	Surveillance and optimization: As part of ongoing quality assurance and performance improvement, mechanisms are in place to monitor, detect, and report on the safety and safe use of HIT and to leverage HIT to reduce patient harm and improve safety

^a^ Adapted from the Health IT Safety framework.^6^

## Methods

### Design and Setting

In this retrospective cohort study, qualitative content analysis was performed to evaluate the role of HIT in RCAs of outpatient diagnostic delays submitted to the VA National Center for Patient Safety (NCPS). The VA health care system provides care to 9 million veterans at 172 medical centers and 1069 outpatient sites using a comprehensive, in-house–designed EHR that since 2000 has been integrated into all facilities.^35^ The NCPS leads patient safety initiatives within the VA health care system and uses RCAs of adverse events and close calls to promote learning across the system. In addition, the NCPS provides extensive training for local patient safety managers responsible for conducting RCAs, emphasizing a focus on systems rather than individual errors, taking into account principles of just culture, human factors engineering, and EHR usability.^36^ Patient safety managers are responsible for assembling the local RCA team, which may or may not include an IT professional. On completion of an RCA, the team presents recommendations to local leadership and submits a detailed report to the NCPS database, including a narrative description of the event, root causes, contributing factors, lessons learned, and action plans designed to prevent future adverse events. Previous aggregated reports highlighted process breakdowns in outpatient diagnostic delays.^14^ Study approval and oversight was provided by the Ann Arbor VA Research and Development Committee. Informed consent was waived because of the use of deidentified data.

### Data Collection

The setting was the US Department of Veterans Affairs. All RCAs categorized by the terms *delay* and *outpatient* and submitted to the VA NCPS from January 1, 2013, to July 31, 2018, were extracted (n = 214) (eFigure in the Supplement). Methods and findings are reported based on the Strengthening the Reporting of Observational Studies in Epidemiology (STROBE) reporting guideline.^37^ We identified RCAs associated with delay in diagnosis (vs treatment or surgery) through manual review (n = 135). One of us (L.P.) reviewed each case in detail to identify only those RCAs involving some aspect of HIT (n = 88) for subsequent detailed analysis. For each RCA, we recorded the diagnosis (if known), diagnostic delay time (ie, time from initial missed finding to diagnosis) if sufficient data were available, and information on diagnostic tests. One of us (L.P.) extracted unique HIT-related safety concerns from each RCA for subsequent coding. We coded safety concerns using content analysis, applying both a deductive approach to code according to the Health IT Safety framework and an inductive approach to allow for emergent codes to provide a richer descriptive analysis and development of subsequent themes.^38,39,40,41,42^

### Qualitative Analysis

To ensure consistent application of the Health IT Safety framework, the first 30 RCAs (involving 77 unique safety concerns) were coded collaboratively by 3 of us (L.P., D.F.S., and H.S.) according to the HIT safety domain that was applicable (safe HIT, safe use of HIT, or using HIT to improve safety) and 1 or more of the 8 sociotechnical dimensions involved (Table 1). Discrepancies were resolved by consensus. After a shared understanding of the approach was achieved through consistent agreement, one of us (L.P.) coded the remaining RCAs, seeking input in complex cases that involved uncertainty in categorization. Throughout the deductive coding process, we also applied open inductive coding to identify emerging patterns. Content of emergent codes was further refined and combined via a collaborative and iterative analysis of the entire data set. Emergent codes were then organized into higher-level themes to identify high-risk areas to target potential solutions. Similar to previous work,^9,43^ we used consensus methods for coding. In addition, because the categories that we coded were not mutually exclusive, we were not able to calculate interrater reliability. However, such consensus methods improve identification of high-risk areas (compared with independent coding) because of multidisciplinary discussions between clinicians, safety experts, and informaticians. Microsoft Excel software (Microsoft Corp) was used to code and analyze the data.

## Results

Of 214 RCAs included, 88 involved HIT-related safety factors in diagnostic delays. Delayed diagnoses involved cancer (n = 55), infection (n = 10), cardiovascular disease (n = 6), and other (n = 5) (ie, 2 diabetic ketoacidosis, 1 testicular torsion, 1 amyloidosis, and 1 benign pancreatic mass). In 12 cases, local site investigators did not report a single final diagnosis and instead described breakdowns in the diagnostic process (eg, loss of >1000 test result letters from a printer malfunction and delays in triage, consultations, and processing laboratory specimens). For cases in which sufficient information was available to calculate diagnostic delay time (n = 69), the median diagnostic delay was 6 months (range, 4 days to 60 months; interquartile range, 2-12 months). In most RCAs involving HIT and diagnostic delays (n = 64), the primary process breakdown involved inadequate follow-up of 1 or more abnormal test results, including imaging (n = 42), laboratory tests (n = 15), and biopsies (n = 8). In one RCA, 2 results were delayed (both an imaging result and a biopsy result).

From 88 RCAs, 172 unique HIT-related safety concerns (mean [SD], 1.97 [1.53] per RCA) were extracted. Table 3 summarizes categorization of the safety concerns according to HIT safety domain and sociotechnical dimension. Twenty-five safety concerns (14.5%) involved problems with safe HIT, primarily issues with hardware and software, clinical content, and human-computer interface. Examples included failure of test results to transmit, equipment malfunction, and issues with upgrades. Most safety concerns (142 [82.6%]) involved problems with safe use of HIT, predominantly sociotechnical factors associated with workflow and communication, people, and a poorly designed human-computer interface. Examples included failure to respond to inbox notifications, lack of EHR proficiency training, and failure to assign surrogates for inbox coverage. There were 5 safety concerns (0.3%) involving using HIT to improve safety through system measurement and monitoring. In all 5, HIT was used to generate a list of high-risk patient test results for follow-up by the clinical team (eg, positive hepatitis C test results and biopsy results), but follow-up and diagnoses were substantially delayed.

**Table 3.** Health Information Technology (HIT)–Related Safety Concerns by HIT Safety Domain and Sociotechnical Dimension

Sociotechnical dimension	No. of Safety Concerns			
	Safe HIT	Safe use of HIT	Using HIT to improve safety	Total
Hardware and software	14	0	0	14
Clinical content	8	8	0	16
Human-computer interface	3	17	0	20
People	0	51	0	51
Workflow and communication	0	61	0	61
Internal organizational features	0	4	0	4
External rules and regulations	0	1	0	1
System measurement and monitoring	0	0	5	5
Total safety concerns	25	142	5	172

During the process of analyzing and coding safety concerns according to HIT safety domain and sociotechnical dimension, several distinct (but not mutually exclusive) themes emerged. These themes were classified into the following 5 high-risk areas associated with diagnostic delays: managing EHR inbox notifications and communication, clinicians gathering key diagnostic information, technical problems, data entry problems, and failure of a system to track test results (Table 4).

**Table 4.** Health Information Technology–Related High-risk Areas in Root Cause Analysis (RCA) Events Associated With Diagnostic Delays

High-risk area	No. of RCAs involved^a^
**Managing electronic health record inbox notifications and communication**	
Notification sent but not acted on^b^	34
Notification fatigue/information overload	20
Inadequate surrogate coverage for staff absence	18
Inadequate system knowledge (new staff or lack of training)	15
Ambiguous responsibility for follow-up	11
Inadequate electronic communication (delayed or miscommunication)	16
**Clinicians gathering key diagnostic information**	
Lack of interoperability (obtaining and viewing outside records)	15
Necessary information difficult to find	14
Patients seen in clinic without review of abnormal test results	13
**Technical problems**	
Notification not generated	15
Malfunctioning radiology codes	3
Notification disappeared on opening	6
Equipment malfunction	2
Hidden dependencies	1
Issues with upgrades	1
**Data entry problems**	
Order entry	16
Missing documentation	4
**Failure of a system to track test results**	
Failure or lack of established tracking system	7
Tracking system eventually followed up on test results, but the diagnosis was still delayed	5

^a^ Numbers refer to how many RCAs involved a particular high-risk area or theme. The RCAs contained multiple non–mutually exclusive high-risk areas. Therefore, the numbers do not sum to 88 RCAs.

^b^ This theme had multiple non–mutually exclusive subthemes (eg, notification not acted on because the assigned surrogate received too many notifications or a new clinician did not receive training in how to manage notifications and thus was overloaded with them). Therefore, the numbers do not sum to 34 RCAs.

### Managing EHR In-box Notifications and Communication

Clinicians rely on the EHR inbox for various types of electronic communication associated with test results, referrals, medication refill requests, patient portal messages, and phone calls. The following 2 notable issues occurred.

#### Notification Sent but Not Acted On

In 1 case, a PCP missed a notification from a specialist to order a mammogram for a patient with abnormal breast examination findings. In another case, a clinician was notified via a note to correct an order but simply signed off the note instead of making the correction. A third clinician processed multiple test results within the EHR inbox all at once, missed a positive stool test result, and thus failed to order a follow-up colonoscopy.

Several factors contributed to failure to act on notifications. The first factor is notification fatigue and/or information overload. One clinician received more than 100 notifications daily, which was associated with notification fatigue and missed test results. The second factor is inadequate surrogate coverage for staff absence. Both overload of covering clinicians and failure to assign coverage for inbox notifications occurred. One covering clinician received more than 200 notifications in 1 day and subsequently missed an abnormal test result. In other cases, no one was assigned to cover staff on extended leave (eg, no one received biopsy results sent to a clinician on maternity leave) or temporary clinicians, such as residents and locum tenens who had left the organization (eg, no one received imaging results sent to a resident who had completed the rotation). Inadequate coverage of nurses, clerks, and coordinators also led to diagnostic delays. The third factor is inadequate system knowledge (new staff or lack of training). Insufficient training contributed to missed notifications, such as when a new clinician did not know how to process notifications efficiently and became overwhelmed. Fourth is ambiguous responsibility for follow-up. A dermatology e-consultant recommended to a PCP that a patient be seen face-to-face in the dermatology clinic. Neither the dermatologist nor the PCP placed the consult order because there was local variation in processes and it was unclear who was responsible to take action. In another case, both the PCP and a specialist were notified of biopsy results, but neither took appropriate action.

#### Inadequate Electronic Communication (Delayed or Miscommunication)

Excessive reliance on EHR documentation for communicating time-sensitive or critical information (eg, through electronic messaging or notes) led to miscommunication and diagnostic delays. In 1 case, a mental health clinician wrote a critical laboratory note to convey important handoff information about a seriously ill patient being transferred to primary care rather than communicating verbally, delaying diagnosis of a life-threatening infection. In another instance, an emergency department (ED) clinician added a PCP as a cosigner on his note but buried abnormal imaging findings in the body of the note rather than in the assessment. The PCP read the note but missed critical information. Other electronic communication problems included using the wrong communication format (eg, placing important information regarding patient symptoms to be triaged in a scheduling tool rather than a triage tool) and relying too heavily on note templates that failed to communicate critical information (ie, low signal-to-noise ratio).

In the context of the Health IT Safety framework, safety concerns in this high-risk area involved mostly problems with safe use of HIT (ie, usability and workflow integration) rather than malfunctions of HIT itself as designed and often involved interactions of multiple sociotechnical domains. For example, 1 case of a missed inbox notification may have been associated with lack of clinician training in how to manage test results (ie, people), too many test results to process (ie, clinical content and workflow and communication), and poor visibility of an abnormal test result (ie, human-computer interface), reflecting the complex characteristics of these safety concerns.

### Clinicians Gathering Key Diagnostic Information

There were several problems with clinicians gathering key diagnostic information. The first problem was a lack of interoperability (obtaining and viewing outside records). Issues with gathering information from both VA and non-VA clinicians included delays in obtaining records, missed fax reports, delays in outside organizations posting diagnostic information to web portals created specifically to share records, and failure to alert clinicians to review scanned records.^44^ The second problem was that necessary information was difficult to find, which often was associated with poor visibility of important data and low signal-to-noise ratio within the EHR. Examples included relevant information buried in hundreds of pages of scanned documents, abnormal findings located in the body rather than impression section of radiology reports, addenda to radiology reports and clinic notes missed because they were at the bottom of the screen, poor visibility of scanned laboratory results, and serious medical conditions buried in clinic notes rather than documented on the problem list. The third problem was that patients were seen in clinic without review of abnormal test results. Even though a patient was seen in clinic multiple times, prior abnormal test results were not reviewed. In the context of the Health IT Safety framework, safety concerns were mostly problems with safe use of HIT, in particular issues with human-computer interface (eg, cluttered screens with poor visibility of important data) and workflow and communication (eg, delays in obtaining records associated with missed fax reports).

### Technical Problems

Technical safety concerns involved 6 problems with safe HIT, mainly malfunctioning hardware and software. The first safety concern was a failure to generate notifications. In some cases, no notifications were generated to cue clinicians to review outside records scanned into the medical record. In another case, the clinician had altered settings so that only abnormal test results would generate a notification. One laboratory test did not have an abnormal cutoff value listed and so was not flagged as abnormal even though it was. The clinician did not receive a notification and missed the test result. The second concern was malfunctioning radiology codes. The use of inactivated radiology codes failed to trigger notifications. The third concern was that notification disappeared on opening. Clinicians lost track of test results if they were interrupted when processing them as notifications disappeared after opening. The fourth concern was equipment malfunction. A malfunctioning printer failed to print more than 1000 test result notification letters. Laboratory processing equipment broke. The fifth concern was hidden dependencies. Orders were inadvertently left active in some places in the EHR when they were deactivated elsewhere. The sixth concern involved issues with software upgrades. For example, recall appointments were lost during an EHR software upgrade.

### Data Entry Problems

#### Order Entry

Subspecialty consultations were discontinued or delayed because of inadequate information in the electronic consultation order. In 1 case, a consultation for a new finding was transmitted to the urology service. However, the consultation was discontinued administratively because the patient was already being followed up in urology for another issue, and the new finding on the consultation order was missed. In another case, even though it was against the organization’s local policy, an ED clinician was able to place a consultation for an outpatient subspecialist directly from the ED, which was subsequently discontinued without the PCP being notified to reorder it. Another problem was the inability to communicate priority for an urgent order because the only categories available were stat and routine, associated with both overuse and underuse of stat for orders with an urgent need. Other issues involved outdated tests listed in order menus and poor visibility of existing decision support to help clinicians order the correct test. Certain cases involved lack of bundling of required orders (eg, a consult order to an outside institution to perform magnetic resonance imaging was not bundled with the required imaging order, allowing a clinician to order the consult but not the imaging).

#### Missing Documentation

Clinic notes were missing, and attempts to notify patients of test results were not documented properly. In the context of the Health IT Safety framework, most safety concerns involved problems with safe use of HIT, primarily associated with human-computer interface (poor design of order entry and decision support), workflow and communication (failure to document attempts to notify patients of test results), and people (missing clinic notes).

### Failure of a System to Track Test Results

Only a few RCAs specifically mentioned problems with systems for tracking test results, although this likely involved most cases of missed test results. In certain cases, failure or lack of an established tracking system was the main safety concern, whereas in others an established tracking system broke down, such as when a melanoma finding was not entered into the biopsy tracking system and when recall software for colonoscopies malfunctioned. In 5 cases, a tracking system eventually followed up on test results (eg, a nurse reviewing a registry noted a positive stool test result and alerted the clinician to order a colonoscopy), but the diagnosis was delayed. Although in certain cases tracking systems were safety nets to eventually help detect missed test results, they were not always widely used, timely, or error-proof. Safety concerns in this high-risk area involved problems with using HIT to improve safety through system measurement and monitoring.

## Discussion

In this retrospective cohort study, outpatient diagnostic delays involving HIT were analyzed, and many sociotechnical problems with safe use of HIT were found, primarily including issues with people (eg, lack of training and failure to act on notifications), workflow and communication (eg, inadequate surrogate coverage and electronic miscommunication), and human-computer interface (eg, order entry design and poorly visible information). Problems involving safe HIT were less common and primarily involved hardware and software and clinical content. Despite the use of test result tracking systems to improve safety, diagnoses were still delayed in a few cases. The following 5 key high-risk areas led to diagnostic delays: managing EHR inbox notifications and communication, clinicians gathering key diagnostic information, technical problems, data entry problems, and failure of a system to track test results.

Study findings confirm the presence of delays in serious diagnoses, including cancers, infections, and cardiovascular disease, because of missed follow-up of test results.^24,25,26,27,28^ Our study builds on prior evidence of high inbox notification burden^29,30,31,32,33^ and suggests harm from diagnostic delays directly attributable to information overload from excessive notifications. RCA data support previous literature highlighting the hazards of inadequate surrogate coverage and ambiguous responsibility in dual-alert communication (ie, notification of both ordering clinician and PCP).^15,45,46^ In addition, although the EHR facilitates asynchronous electronic communication between clinicians through both electronic messaging and note-based communication, this discouraged verbal communication in several situations and increased reliance on EHR templates, with subsequent risk of misunderstanding.^10,12^ Application of the Health IT Safety framework suggests that many problems with diagnostic delays described herein were associated with usability, design, and workflow integration.

Analysis of aggregated RCAs provided meaningful information even though experts have recently questioned the value of RCA investigations for improving patient safety.^47,48,49^ Experts point to reasons like the singular focus on finding the root cause, questionable quality of investigations, hindsight bias, poorly functioning feedback loops, and failure to aggregate learning across incidents.^47^ Rather than implementing design or structural changes, many RCAs suggest weak actions, such as additional training and policy reinforcement, which are unlikely to decrease event occurrence.^48,49^ We attempted to overcome these limitations by aggregating analysis across the entire VA health care system. Such aggregate analysis of similar types of patient safety issues is rarely done at an individual organization level but is useful to focus attention on common and broader themes (Table 5) invisible to local site investigators,^50,51^ who tend to focus on weaker interventions, such as policy reinforcement and training, rather than high-level system changes with larger consequences.

**Table 5.** High-risk Areas and Suggested Interventions for Health Information Technology–Related Diagnostic Delays

High-risk area	Suggested interventions
Managing electronic health record inbox notifications and communication	Reduce burden of notifications by eliminating unnecessary messages Streamline communication by highlighting important information Train new clinicians how to efficiently and effectively process notifications Ensure adequate surrogate coverage Delineate clear lines of responsibility Design inboxes to better sort messages and highlight important information
Clinicians gathering key diagnostic information	Strive for interoperability with outside facilities Improve visibility of critical information Create tools/interfaces to assist in information gathering and data visualization Include clinicians in electronic health record user interface design
Technical problems	Ensure adequate information technology resources to report and fix technical issues rapidly Ensure downtime and reactivation procedures are clear and used as needed Improve data interoperability reliability
Data entry problems	Streamline and optimize order entry for tests and consult requests Ensure adequate documentation but use required data entry fields judiciously
Failure of a system to track test results	Develop safety nets for tracking high-risk test results to ensure timely follow-up Assign staff to regularly review electronic tracking systems Identify inappropriate and unsafe workarounds used to manage test results

Although it appears that a large number of safety concerns were associated with people using HIT, these cannot be considered as faults of the individuals involved. Cognitive lapses often occur even when the EHR is used as designed and are symptoms of broader system problems with clinical and administrative workflows and EHR design. A poorly designed system increases cognitive demands on individuals and heightens opportunity for human error. This complex interplay between human cognition and the system is well recognized within the discipline of human factors, including the “application of what we know about people, their abilities, characteristics, and limitations to the design of equipment they use, environments in which they function, and jobs they perform.”^52^ Therefore, interventions to reduce diagnostic delays will need to draw on principles from human factors engineering to design the EHR and work system so that it provides clinicians with the cognitive support they need to do their jobs.

Several interventions could address this multifactorial problem. The first intervention is to redesign EHR inboxes and message workflow. The EHR inboxes could be redesigned to better prioritize, display, and sort messages; track high-risk test results; and allow messages to be easily reassigned to support staff to reduce overload.^53^ Several recommendations for improvement exist, such as increasing message processing efficiency and decreasing clicks, redesigning the inbox interface, reducing cognitive load, and limiting duplicate or low-value messages.^53^ All clinicians should be competent in optimal test result management strategies that increase efficiency and decrease errors.^54,55,56^ Adequate inbox coverage should be ensured for clinicians who are out of the office or have recently left the organization.^45^ Efforts should be made to reduce the number of inbox notifications.^57^ New initiatives that rely on sending additional notifications to clinicians who are already overwhelmed should be avoided. Electronic communication could be streamlined to include only relevant information, and “FYI,” low-value, and duplicate communication should be minimized.^32^

The second intervention is to develop safety nets to identify missed test results. One example is Kaiser Permanente’s SureNet system to identify test results that still need action.^58,59,60^ Electronic trigger tools have been developed to selectively identify missed test results that have not received expected follow-up actions, and additional development and implementation could address diagnostic delays in high-risk conditions, such as cancer.^26,61^ These innovations are already being tested in the VA health care system. Encouraging patients to access test results directly through online portals may provide another safeguard.^62^

The third intervention is to improve display of diagnostic information. Research should focus on improving usability of interfaces that are difficult to use or those that obscure important patient information.^8,63,64,65,66,67^ Clinicians should be included in user interface design processes that strive to improve visibility of critical information and facilitate more efficient information review.^68^

The fourth intervention is to track referrals. Organizations should develop tracking systems for electronic specialty referrals to reduce breakdowns in the referral process and “close the loop” to referring clinicians.^69^

The fifth intervention is to optimize order entry design. Order sets could be redesigned to provide helpful, noninterruptive decision support and automatically pull in required information rather than relying on manual clinician entry for basic information.^70^ Adequate IT resources are needed to report and fix technical issues expeditiously.

The sixth intervention is to pursue interoperability. Lack of interoperability limits the availability of diagnostic information when patients transition care to a new clinician.^71^ Pursuing interoperability between different VA and non-VA community settings could improve access to important diagnostic information and reduce diagnostic delays. The recent Office of the National Coordinator for Health Information Technology’s Cures Act Final Rule is a step in the right direction.^72^

### Limitations

This study has several limitations. All incidents involved the use of different configurations of the same EHR within a single, large, geographically distributed delivery system and might not be generalizable to other EHRs or health systems. However, other EHRs also have inbox–like notification mechanisms,^73,74^ electronic communication of test results,^46^ electronic referrals,^75^ and screen designs that are difficult to use.^76,77^ Evidence of order entry problems, diagnostic information that is difficult to find, information overload, and limited physician time to process EHR notifications has emerged from other health systems.^67,78,79,80^ Although our sample size was small, case descriptions were rich, spanned a period of 5 years, and involved multiple geographic locations across the United States. In addition, our sample was limited by voluntary reporting and may not be representative of all types of diagnostic delays. Although all events had high actual or potential for harm ascribed to them by local safety personnel, assigning harm, particularly potential harm, is subjective. Most safety personnel are not specifically trained to evaluate EHR usability, and some usability issues may have gone undetected. Indeed, our findings of predominantly people and workflow problems may reflect the tendency of RCA teams to complete their analysis after identifying a human error rather than digging deeper into system and design problems.^47,48,49^ Finally, reports are biased because of voluntary reporting, with no controls or noncases for comparison, and do not reflect the true underlying epidemiology of these errors. Nevertheless, cases identified high-risk areas associated with diagnostic delays that can be further explored in epidemiologic studies.

## Conclusions

In this qualitative content analysis, the Health IT Safety framework was used as a lens to identify several high-risk areas in outpatient diagnostic delays, many of which are applicable to other health systems using EHRs. These aggregated RCA data provide evidence that high-yield interventions could be aimed at improving test result management, interoperability, data visualization, and order entry, as well as reducing information overload and overreliance on electronic documentation for communicating critical information. The complexity of the association between HIT and diagnostic delays described herein underscores the need for collaboration between clinicians, health system leaders, safety professionals, and HIT designers in the testing and implementation of interventions to improve outpatient safety.

## Notes

Supplement. **eFigure.** Data Collection and Analysis
