# Supplementary material for: Assessment of Health Information Technology–Related Outpatient Diagnostic Delays in the US Veterans Affairs Health Care System: A Qualitative Study of Aggregated Root Cause Analysis Data
Source: JAMA Netw Open. 2020 Jun 25;3(6):e206752. doi: 10.1001/jamanetworkopen.2020.6752 (PMC7317596; doi:10.1001/jamanetworkopen.2020.6752)
Supplement: Supplement. — eFigure. Data Collection and Analysis [file jamanetwopen-3-e206752-s001.pdf]

Supplementary Online Content

Powell L, Sittig DF, Chrouser K, Singh H. Assessment of health information technology–related outpatient diagnostic delays in the US Veterans Affairs health care system: a qualitative study of aggregated root cause analysis data. *JAMA Netw Open*. 2020;3(6):e206752. doi:10.1001/jamanetworkopen.2020.6752

eFigure. Data Collection and Analysis

This supplementary material has been provided by the authors to give readers additional information about their work.

© 2020 Powell L et al. *JAMA Network Open*.

eFigure. Data Collection and Analysis

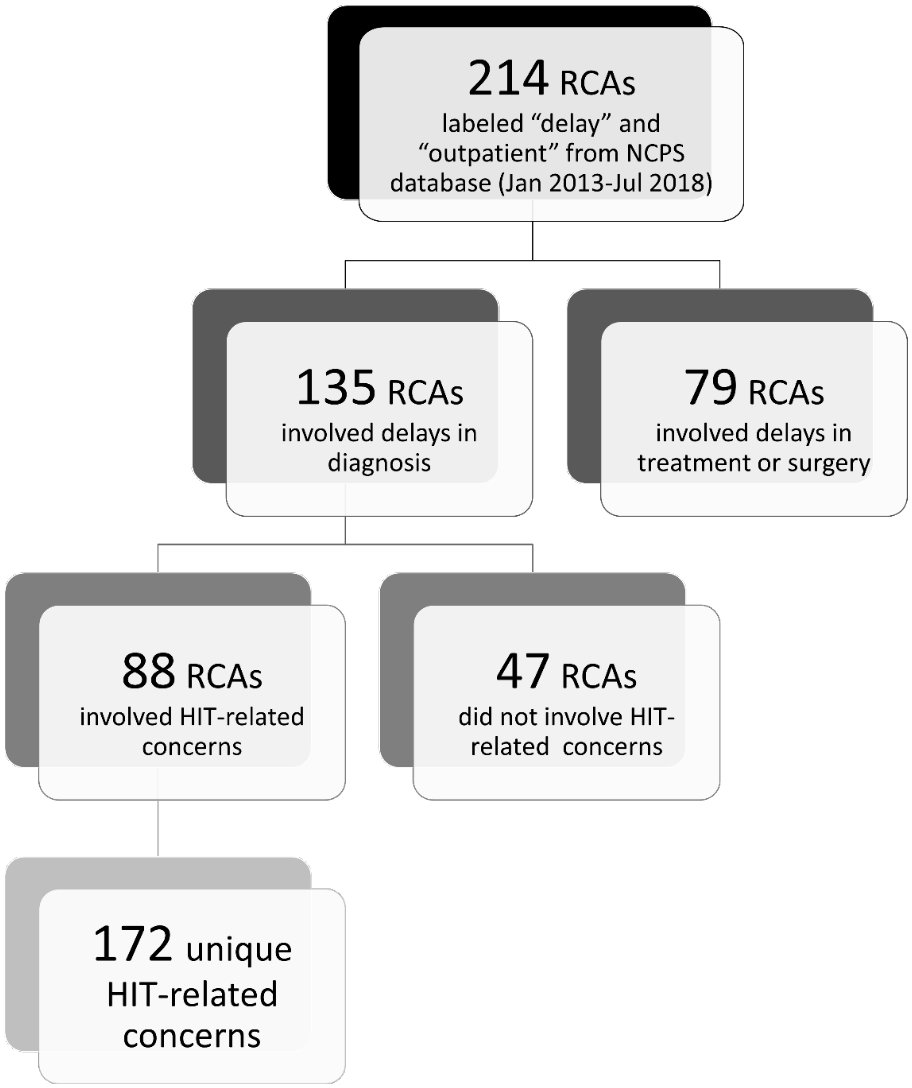

NCPS = National Center for Patient Safety; RCA = root cause analysis; HIT = health information technology

© 2020 Powell L et al. *JAMA Network Open*.
